# Assessing the Effectiveness of Neurofeedback Training in the Context of Clinical and Social Neuroscience

**DOI:** 10.3390/brainsci7080095

**Published:** 2017-08-07

**Authors:** Franklin Orndorff-Plunkett, Fiza Singh, Oriana R. Aragón, Jaime A. Pineda

**Affiliations:** 1 Department of Electrical Engineering, University of California, San Diego, La Jolla, CA 92093, USA; forndorf@eng.ucsd.edu; 2 Departments of Psychiatry, University of California, San Diego, La Jolla, CA 92093, USA; fizasinghmd@gmail.com; 3 Marketing Department, Clemson University College of Business, Clemson, SC 29634, USA; oriana.aragon@gmail.com; 4 Department of Cognitive Science, University of California, San Diego, La Jolla, CA 92093, USA; 5 Neurosciences Group, University of California, San Diego, La Jolla, CA 92093, USA

**Keywords:** neurotherapies, perturbative physiological plasticity, self-directed plasticity, biomarkers, functional connectivity

## Abstract

Social neuroscience benefits from the experimental manipulation of neuronal activity. One possible manipulation, neurofeedback, is an operant conditioning-based technique in which individuals sense, interact with, and manage their own physiological and mental states. Neurofeedback has been applied to a wide variety of psychiatric illnesses, as well as to treat sub-clinical symptoms, and even to enhance performance in healthy populations. Despite growing interest, there persists a level of distrust and/or bias in the medical and research communities in the USA toward neurofeedback and other functional interventions. As a result, neurofeedback has been largely ignored, or disregarded within social neuroscience. We propose a systematic, empirically-based approach for assessing the effectiveness, and utility of neurofeedback. To that end, we use the term perturbative physiologic plasticity to suggest that biological systems function as an integrated whole that can be perturbed and guided, either directly or indirectly, into different physiological states. When the intention is to normalize the system, e.g., via neurofeedback, we describe it as self-directed neuroplasticity, whose outcome is persistent functional, structural, and behavioral changes. We argue that changes in physiological, neuropsychological, behavioral, interpersonal, and societal functioning following neurofeedback can serve as objective indices and as the metrics necessary for assessing levels of efficacy. In this chapter, we examine the effects of neurofeedback on functional connectivity in a few clinical disorders as case studies for this approach. We believe this broader perspective will open new avenues of investigation, especially within social neuroscience, to further elucidate the mechanisms and effectiveness of these types of interventions, and their relevance to basic research.

## 1. Introduction

A goal of social neuroscience is to draw causal conclusions about neural activity in relation to social behavior, thought, perception, and experience. Methodological strategies oftentimes manipulate external factors, or participants’ social experiences, to observe corresponding neural activity. Alternatively, neural activity might be considered a treatment variable, with experimental manipulation of targeted activity. This has been accomplished in a variety of ways, including transcranial magnetic stimulation (for example, see [1]), but this might also be accomplished via neurofeedback. Consideration of neural activity as a factor rather than an outcome variable widens the breadth of possible research, and warrants consideration by the social neuroscience community.

To date neurofeedback has been applied in the treatment of psychiatric pathology such as autism spectrum disorders [2], attention-deficit/hyperactivity disorder [3], and depression [4]. However, the principles of neurofeedback have also been applied to nonclinical populations, for example in targeting attentional focus, memory [5,6], and emotion regulation [7]. As much as social neuroscience is suggested to be able to illuminate psychiatric pathology [8], neurofeedback methodology, once considered confined to clinical treatment, may offer alternative options in experimental research when designed with full ethical considerations.

Neurofeedback training can range from a single-session manipulation to a lengthy process ongoing for months in pursuit of enduring changes. The efficacy of neurofeedback training can be measured both in changes to baseline neural activity, but also in manifested outcomes of social behavior, thought, perception, and experience. While neurofeedback has begun to be used in fMRI experiments (for review, see [9]), there are far fewer experimental designs using electroencephalography (EEG) neurofeedback, perhaps because of a lack of systematic approaches to assess the methodology. Here we propose a systematic, empirically based approach for assessing the effectiveness, and utility of EEG neurofeedback. Such a framework provides footing for the evaluation of neurofeedback and its application in clinical as well as social neuroscience research.

### 1.1. The Goal of Neurofeedback

At its most fundamental level, neurofeedback training provides learners with information about their current behavioral, physiological, and neural processes in the service of self-directed modulations of those processes to achieve specific outcomes. In early uses of feedback, such as biofeedback, in which learners were provided with their current physiological state, they were asked to meet a goal, such as autonomic balance [10,11]. Neurofeedback, a derivative of biofeedback, has been used in clinical practice since early experiments conducted by Kamiya in the 1960s [12,13] and Sterman in the 1970s, in which epileptic cats were trained to enhance a sensorimotor brain rhythm, leading to remarkably reduced susceptibility to epilepsy [14].

As described by many of the clinicians and researchers who pioneered these fields, feedback techniques allow participants to monitor, interact with, change, and manage their physiological and mental states. In fact, the clinical viability and enthusiasm inherent in this field lies in promising results, i.e., identification of various physiological markers, such as heart rate variability, peak alpha frequency, and skin conductance that can be self-regulated and whose regulation corresponds with improved health, along with targeted behavioral and psychological outcomes. Typically, bioelectric signals are digitized and displayed for the user and practitioner, and provide an evidence-based methodology that is applied to a variety of psychiatric problems, including attention deficit hyperactivity disorder (ADHD), bipolar disorder, schizotypal personality disorder, dissociative identity disorder, major depressive disorder, post-traumatic stress disorder, addiction, and others [15,16]. A significant number of practitioners also use these approaches for “sub-clinical” populations who seek “peak performance” enhancements, citing detrimental issues such as “brain fog,” trouble with energy, and lack of focus [17,18,19].

The scope of neurofeedback application has recently been expanded to include control of hemodynamic (e.g., blood oxygen level dependent (BOLD) signal or hemoencephalographic (HEG) signal) and magnetic fields. Unlike EEG-based neurofeedback, which relies upon electrical activity recorded from the scalp, functional magnetic resonance imaging (fMRI), functional near-infrared spectroscopy (fNIRS), and magnetoencephalography (MEG) neurofeedback provide more precise localization and modulation of relevant brain structures [9,20]. Individuals undergoing training learn to volitionally control these new signals rapidly at a pace similar to the EEG approach [21]. Whereas EEG-based interventions frequently require multiple sessions (e.g., up to several tens of sessions depending on the type of approach and severity of the problems; [15], in real time (rt) functional magnetic resonance imaging (FMRI) neurofeedback individuals learn to modify the BOLD signal within a single 30-min session of training [22,23,24]. Although this is similar to some EEG-based single-channel protocols in which learning was shown to occur within a single session [25], in this respect the rtFMRI approach provides pragmatic advantages comparable to EEG-based methods. In other ways rtFMRI has pragmatic disadvantages, such as cost, loudness, and the overall, closed environment of MRI scanners, as well as the loss of high-definition temporal resolution relative to EEG. It should be noted that the temporal resolution of EEG is an advantage over rtFMRI, given that the fast feedback response, and thus temporal contiguity, is crucial for optimal learning [9]. The availability and unique advantages of rtFMRI and EEG neurofeedback is creating a renaissance of interest.

From a clinical perspective, some contend that these approaches are successful based on the size of the industry and the large numbers of clinicians who use and promote them. A significant number of case reports detail marked improvements, and more formal studies indicate positive group outcomes for a wide variety of neurotherapeutic approaches [26,27,28,29,30,31,32,33,34]. The fact that the industry as a whole has not diminished—and indeed is growing—suggests that many believe in its efficacy. Nonetheless, recent reviews have cast some doubts on the effectiveness and efficacy of feedback interventions (e.g., [35]), citing a number of problems such as the lack of appropriate control conditions or effective double-blind protocols. While many clinicians show support for NFT, for some, concerns about untrustworthiness and even bias in the medical and research communities has created problems. This has often meant that neurofeedback training is discounted or disregarded as a therapeutic option.

Our goal in this chapter is not to review the extensive neurofeedback literature but rather to provide the rationale for an organizing principle that might recast the assessment of the efficacy of these techniques. We do so by examining the criteria by which such interventions are judged effective. We look at current trends and progress in neurofeedback studies that provide evidence for structural and/or functional measures of network connectivity. The working assumption is that neurological networks mediating specific functions can be modified and strengthened via structural, spectral, and metabolic neuroplastic changes. In particular, measurable changes in information flow or the coherence between regions, or structural changes in white matter tracts triggered by neurofeedback can lend support to the effectiveness of the intervention.

### 1.2. Methodologies

EEG-based methods remain the primary and most often used approach in the delivery of neurofeedback, mainly because of their low cost, non-invasiveness, and ease of use [36]. A variety of such techniques can address a user’s desire for improved cognitive efficiency, recall, performance under pressure, and leadership. Often clinicians use techniques that address specific pathologies in a more rigorous “over-training” manner to bring the patient from a deficit to “healthy/normal” and even towards “exceptional” behavior [21,36,37]. Practitioners use specific techniques like alpha/theta ratios, deep-state training, and synchrony training as a type of self-improvement, psychological centering, or even spiritual practice [38,39,40,41,42].

It is important to begin our discussion by briefly providing examples of some of the modalities in the diverse world of neurofeedback techniques, where researchers have developed distinct protocols that target different brain signals and their concomitant physiological processes [9,43]. Major protocol categories exist that have evolved in a generational fashion beginning with alpha brainwaves and reaching to real-time network-based methodologies. Broadly speaking, these protocols can be placed into categories based on the signal and/or the focus of training: (1) basic frequency training; (2) deep-state or hypnogogic training; (3) infra-low frequency (ILF) and slow cortical potential (SCP) training; (4) synchrony/cross-frequency coupling; (5) network-based training; and (6) normative and symptom-based selection. It is beyond the scope of this chapter to explore all of these methods in depth; rather we choose to look closely at those that were developed in a clinical setting with the idea of training the connectivity and overall integration of the brain.

#### 1.2.1. Frequency Training (δ,θ,α,β,γ)

In basic frequency training, the EEG placement typically includes a few electrodes at target sites (according to the International 10–20 system), a ground connection, and a reference. Processing of signals requires a digital filter to isolate rewards and inhibit frequencies, e.g., one might design a protocol in which increased spectral power in the sensorimotor rhythm or upper alpha are presented to the participant as reward “hits” or a combination of low theta with high beta are presented to participants as reward “misses.” The basis of this protocol is to address an identified deficit or excess of a particular frequency band relative to a set of bands observed in the subject or patient at initial intake. In clinical practice, if a particular band at a specific electrode site has low power based upon the clinician’s assessment (either from a normative evaluation using quantitative EEG (QEEG) or symptom-based evaluation that references protocols with a history of success), the clinician will design a protocol to exercise the power output at that site. Activity thresholds (i.e., the spectral power of the aforementioned rewards and inhibits) are established for individual sites and different frequency bands. Progress and contraindications are largely subjective and based on the clinician’s experience, except for the case of a QEEG, and are evaluated qualitatively as the client proceeds through treatment sessions. The neurofeedback literature produced in academia has been more rigorous in design and exacting in determining the contraindications and progress of subjects who take part in neurofeedback sessions. Among the most well-studied frequencies are alpha (8–12 Hz), upper alpha (10–12 Hz), sensorimotor rhythm (SMR 12–15 Hz), and gamma (~35–45 Hz) [21,36,37].

#### 1.2.2. Deep State or Hypnogogic Training

Although alpha/theta (α/θ) ratio protocols were established in the late 1970s, they remain remarkably unchanged from their original form and are still widely used in clinical settings to address affect disturbances, behavioral problems, and dysregulation, especially in cases of post-traumatic stress. Although there is as of yet no consensus regarding the mechanisms and origins of alpha oscillations, they have been studied extensively [44,45,46,47]. Alpha/theta protocols capitalize on the emergence of alpha dominance—the so-called Berger alpha rhythm, named after Hans Berger, the inventor of EEG, who discovered this activity—that generally occurs posteriorly when the eyes are closed. Gruzelier [48] cited several studies indicating that theta oscillations play a large part in long range functional interaction and especially in working memory [49]. The overall working principle for deep-state training is the α/θ bridge, or the spectral zone of integration of information between α and θ bands corresponding to conscious and subconscious processes, respectively [48,50,51,52,53].

Theta rhythms (4–7 Hz band) are oscillations that have been shown to play important roles in subcortical and mesencephalic regions, broadly categorized as the limbic system. At the level of cognition and behavior, they have been implicated in many processes and mental states [48,54,55,56,57,58]. The subcortical systems from which theta rhythms arise are known to serve regulatory control of arousal, affective state, learning, and memory as well as cognitive and attentional focus [48,59]. As theta oscillations are associated with such a wide variety of neurological and psychological functions, this suggests that theta-based neurofeedback would influence a similarly diverse set of cognitive processes.

From this cursory understanding of the psychophysiological significance of alpha and theta frequencies, alpha/theta neurofeedback protocols have been designed to ease the client into a state during which he or she makes brief excursions from an alpha into a predominantly theta state while maintaining awareness. A “crossover” occurs in which theta power supersedes alpha power, while maintaining low delta activity to avoid sleep, and low beta to avoid inducing anxiety. This crossover state is associated with reduced anxiety, drowsiness, sleep onset, and, most importantly, re-experiencing biographical episodes [52,60]. The alpha/theta protocol is hypothesized to bridge cortical and neo-cortical activity and loosely subcortical and basal activity to allow for integration and reprocessing of traumatic experiences in post-traumatic stress disorder [52,53]. It is also a protocol that has been used as a peak-performance enhancer of creativity [61].

#### 1.2.3. Infra-Low Frequencies and Slow Cortical Potentials

Several developments in low-frequency neurofeedback have been introduced recently. Although they have their origins in traditional higher frequency training, infra-low frequency training (ILF) and slow cortical potentials (SCP) neurofeedback in the range of 0.0001–0.01 Hz are two slightly different methodologies in a similar spectral regime. SCP, developed largely by researchers at the University of Tubingen [62,63,64], involves operant conditioning training to an upper and then a lower threshold, with the goal of gaining control over slow cortical potential activity, and thus flexibility and control of neuronal activation and regulation in macro-regions of the brain. ILF is intended for the subject or patient to witness and “attune” to fluctuations in their own neuronal activations without expending a great deal of effort. Physiologically, low-frequency electrical activity has been implicated in glial function, overall local neuronal activation, and metabotropic state [16,65,66]. Birbaumer and colleagues [63] have shown that these slow varying potentials play a role in neuronal clusters’ preparation to fire.

Low-frequency training regimens have been used for more than a decade to address issues related to post-traumatic stress disorder, attentional problems, and anxiety disorders [67]. Even complex issues that remain largely under-investigated such as attachment resolution, complex PTSD, and behaviors associated with personality disorders have been addressed [68]. While the overall working mechanisms of ILF and SCP training are not fully understood, including the implications of how such training may influence metabolic or endocrine function, or potentially even transcriptional regulation at the receptor level, their apparent success and widespread use in the clinical community require further study.

#### 1.2.4. Synchrony, Coherence and Cross-Frequency Coupling

Ideas regarding phase synchrony, coherence, and cross-frequency coupling arose from observations regarding interactions between spatially separate regions and spectrally separate oscillations in the frequency domain. Synchrony and coherence, though related, have distinguishing features. Conceptually, synchrony, or, more commonly phase synchrony, addresses phase relationships between the narrow band-pass filtered signals from two sites, whereas coherence addresses the overall correlation of these two signals, including information about both phase and amplitude. In addition, coherence is fundamentally a bidirectional measurement, whereas phase synchrony can be better described as a sort of “directed coherence” measurement of the flow of information [69]. Mathematically, these are usually described as statistical processes, the instantaneous phase difference (Equation (1)) and correlation coefficient (Equation (2)) of two band-passed signals *x* and *y*, where the *^* signifies zero-mean, normalized transformations of the original signals. Both of these measures have been used to correspond to communication between brain regions [69,70]. Protocol designs related to coherence and synchrony training have been developed based on the idea that specific coherence and synchrony deficits or excesses are correlated with specific behavioral, cognitive, or affective problems [71,72].

(1) $$\Phi_{xy}\left(t\right)=\left|n\phi_{x}\left(t\right)-m\phi_{y}\left(t\right)\right|\;\;\;\;\;\;\left(Eq\;1\right)$$

(2) $$C_{xy}\left(\tau \right)=\int_{u}\hat{x}\left(u\right)\cdot \hat{y}\left(u-\tau \right)du\;\;\left(Eq\;2\right)$$

Physiologically, phase synchrony commonly arises in lower frequencies, namely delta, theta, and alpha, for each originates from separate and unique subcortical generator nuclei, including the thalamus and hippocampus. In task-related processing, i.e., when the brain is not at rest, these rhythms are thought to desynchronize. Conversely, in the resting state, as a general rule, there exists a state of synchrony. Thus, when an individual’s eyes are closed and the visual system is not engaged in processing sensory information, posterior alpha activity becomes synchronized and increases in power. Neurotherapeutic approaches in lower frequency domains are based on this idea and attempt to train the brain to relax and idle at the subcortically generated alpha rhythm without engaging and desynchronizing. As [73] described in 2004, synchrony is less metabolically demanding.

Cross-frequency coupling (CFC), in contrast to synchrony and coherence, involves interactions between spectral regions at specific sites. This class of neurofeedback is new territory and less well established in the literature. These interactions come in several forms that will not be fully discussed here; more extensive treatments can be found in a number of recent reviews (for example, [74]). As a new metric in clinical EEG, CFC has been found to be important in information processing and interregional communication within the brain, correlated with fluid intelligence and perception. Canolty and Knight [75] have described CFC as the framework “to transfer information from large-scale brain networks operating at behavioral timescales to the fast, local cortical processing required for effective computation and synaptic modification, thus integrating functional systems across multiple spatiotemporal scales.” Jirsa and Müller [76] have similarly supposed that CFC “plays a crucial role in the organization of large-scale networks and functional integration across long distances.”

CFC EEG biofeedback techniques are still in their infancy and studies have yet to be published. One such NFT protocol is based on the simple case of amplitude CFC and called TAG Sync. The idea is to reward, or at least notify, the client when the envelopes of theta and gamma, and then alpha and gamma, coincide in terms of polarity and overall shape (Larsen 2012). This is to train the client to “nest” more gamma oscillations on theta frequencies as a carrier. These protocol designs, as well as more mathematically sophisticated techniques developed by others (Applied Neuroscience (Largo, FL, USA), developer of NeuroGuide: [77]; and Neurofield Inc. (Bishop, CA, USA), developer of Brainworks: [78]), are used with client presentations involving complex large-scale integration problems.

#### 1.2.5. Normative vs. Symptom-Based Protocol Selection

Different approaches to neurofeedback training have evolved over its history. Arousal theory and system-based neurofeedback protocols were some of the earliest approaches. Changes in arousal levels were assumed to help patients become more balanced. Although such approaches appeared to work for many, there were design issues, such as the lack of proper controls and a lack of rationale for which type of protocol would be used and when. This haphazard approach changed considerably with the advent of quantitative EEG (QEEG). In QEEG, the features of a patient’s individual EEG are quantified using Fast Fourier Transformation (FFT), e.g., power in the alpha band, which is then compared to the same measure in a normative database. A comparison of the patient’s score relative to this normative database allows for the computation of a standard deviation from the mean or z-score, which can then help drive the training [79,80]. Because QEEG-neurofeedback protocols are more data-driven, a host of different approaches have emerged (Live Z-score, LORETA, BrainAvatar, Low-Energy Neurofeedback System [16,81]). Furthermore, the increased evidence for subtypes or subgroups of patients makes the need for prior QEEG measurements almost a necessity. Indeed, evaluation of raw EEG and QEEG data to identify phenotypes, considered stable patterns within the EEG, is a more comprehensive approach to “yield the best treatment outcome according to the phenotype of the individual EEG” [82].

The availability of z-scores from QEEG analyses has led to a recent approach called “real-time z-score neurofeedback.” In this case, thresholds are not set by the therapist but occur as a function of the z-score [83]. That is, frequencies or features such as coherence are either up- or down-trained to approximate the normal score (or at least kept within 0.5–1 SD of normal). Another more recent training protocol involves low-resolution electromagnetic tomography (LORETA) training [84]. In this QEEG-based analysis, one can estimate the location of the underlying neural generators producing the EEG activity. This allows the therapist to target specific underlying areas of the brain for training and can lead to a reduced number of required sessions or provide a different approach for challenging cases that do not respond to other protocols.

#### 1.2.6. Combined EEG and Real-Time fMRI

Imaging methods combining EEG and fMRI date back to the late 1990s. A search of the recent neurofeedback literature shows that since 2011 an increasing number of articles have been published on real-time (rt)-fMRI neurofeedback and its combination with EEG approaches. Unlike most other EEG neurofeedback techniques that rely on electrical activity near the cortical surface, rt-fMRI-NF has the potential to access specific subcortical regions, and in principle any region that can be monitored using BOLD measures, such as the cerebellum, brainstem, and spinal cord. While rt-fMRI NF is a relatively new technique, in a meta-analysis of 12 such studies, Sulzer et al. [9] concluded that successful regulation of neural activity is indeed possible in various brain regions. In fact, their results suggest the possible existence of a ”neurofeedback regulation network” consisting of the anterior insula, basal ganglia, dorsal parts of the parietal lobe, and extending to the temporo-parietal junction, anterior cingulate cortex (ACC), dorso-lateral prefrontal cortex (dlPFC), ventrolateral PFC, and visual areas. Furthermore, successful regulation of the BOLD signal via neurofeedback has shown positive effects on a number of disorders, including schizophrenia [85], depression [86], and nicotine addiction [23]. Nevertheless, in a recent review as part of the Neurofeedback Evaluation & Training group of the French Association of Biological Psychiatry and Neuropsychopharmacology (AFPBN), Arns et al., [87] concluded that the level of evidence for efficacy remains too weak to justify rt-fMRI clinical use, with the possible exception of the treatment of attention-deficit/hyperactivity disorder (ADHD) in children.

In general, neurofeedback approaches rely on a single brain imaging modality such as EEG or fMRI. Combining these modalities for neurofeedback training has been hypothesized to provide richer information to the subject, enabling participants to achieve faster and more specific self-regulation [24,35]. While there are only a handful of combined EEG and fMRI neurofeedback studies, in a recent study comparing unimodal versus multimodal approaches, [9] used a simultaneous EEG-fMRI protocol in which participants performed a motor imagery task. The results showed that motor activations, as revealed by offline fMRI analysis, were stronger during the combined EEG and fMRI-neurofeedback than during the individual EEG neurofeedback.

## 2. Brain Plasticity

All the EEG-based approaches described above are predicated on changing the dynamics of brain activity to produce beneficial and long-lasting changes in function and structure. That is, these neurotherapies take advantage of the inherent neuroplasticity of the brain. In a review on plasticity, Pascual-Leone and colleagues [88] stated that “it is not possible to understand normal psychological function or the manifestations or consequences of disease without invoking the concept of brain plasticity.”

### 2.1. What Is Neuroplasticity?

Neuroplasticity refers to the intrinsic dynamic and time-sensitive ability of the central nervous system (CNS) to change and adapt structurally and functionally in response to environmental pressures, physiological changes, and experiences. It does not represent a temporary state of the organism but an ongoing state of change that lasts a lifetime and occurs at all levels, including the genetic, molecular, synaptic, cellular, and system levels, culminating in a gain or loss of a behavior or function. Neuroplasticity appears to manifest through diverse processes [89], and its nature makes it likely that the outcome may produce either wanted or unwanted behavior. Of specific interest to this review is adaptive plasticity [90], which refers to experience-dependent changes following intense motor skill training, although this phenomenon could apply to complex cognitive/affective states and processing. Experience-dependent structural synaptic plasticity is common throughout the CNS and thought to represent the neurobiological substrate for learning and memory formation. This type of plasticity assumes that change is a direct result of new neural pathways that form in response to your learning efforts. At the same time, throughout development the brain is undergoing “synaptic pruning,” i.e., the elimination of the pathways one no longer needs.

Implicit in this view of plasticity is the notion that any change would be difficult to measure “because any event falls upon a moving target, i.e., a brain undergoing constant change triggered by previous events or resulting from intrinsic remodeling activity.” Likewise, plasticity at the neural level need not necessarily lead to perceptible changes at the behavioral level, and indeed may lead to changes demonstrable only under special testing conditions [88]. Despite these difficulties, sufficient evidence suggests that it is possible to guide this process, suppressing changes that may lead to undesirable outcomes and promoting or enhancing those that provide a benefit to the subject or patient.

Neurofeedback is a collection of techniques for the specific self-regulation of neural dynamics, based on operant conditioning principles. It is hypothesized that it can activate self-regulatory responses that can lead to normalization of abnormal patterns, which originally may have resulted from deviant neuroplastic processes. Although changes in behavior may be a downstream outcome, initially the focus is on change at the level of brain activity itself.

### 2.2. Perturbative Physiologic and Self-Directed Plasticity

In order to capture the unique nature of plasticity-induced changes via neurofeedback, we coin the term perturbative physiologic plasticity (PPP). We describe this as a type of activity-dependent synaptic plasticity and Hebbian learning that is initiated unintentionally by internal or external events. It perturbs the dynamics of the system and can guide it to either wanted or unwanted change. A variation of PPP that is linked perhaps more closely to neurofeedback is self-directed plasticity, which is initiated intentionally or volitionally to perturb the dynamics of the system and guide it from abnormal to normal functioning. In a recent study, Ros et al. [91] demonstrated that half an hour of voluntary control of brain rhythms is sufficient to induce a lasting shift in cortical excitability and intracortical function. These after-effects are comparable in magnitude to those observed following interventions with artificial forms of brain stimulation involving magnetic or electrical pulses. These researchers have also argued that control of network oscillations is unlikely to be epiphenomenal and can induce neuroplastic changes that may lead to changes in cortical function that outlast their phase of entrainment. Thus, control of brain oscillations could be the mechanism harnessed intentionally to mediate plasticity. Of special interest, then, would be to address pathologies in which network connectivity has been shown to diverge from a healthy normal state, such as might occur in autism, schizophrenia, and psychopathy.

## 3. An Organizing Principle for Determining Effectiveness

Starting in the 1960s, EEG-based neurofeedback became a treatment vehicle for a host of mental disorders. However, its mechanisms of action and clinical effectiveness have remained controversial. Indeed, recent accumulating evidence seems to refute the clinical superiority of feedback training over sham treatment [35,92,93,94,95,96]. This evidence argues that even in the best controlled studies involving double-blind, multiple single-blind, and sham-controlled studies, influences other than the feedback itself (e.g., cognitive strategies, placebo, and non-specific factors) bring about improvements in clinical end-points across a range of disorders [35]. It is acknowledged that although a majority of neurofeedback studies, especially those conducted by individual clinicians, have not met rigorous standards in terms of control conditions, sufficient power to make strong statistical claims, etc., the recent trend, primarily from university-based studies, is producing better science. However, there is one area that we believe has remained unexamined in these camps, and that is the question of how one defines efficacy and effectiveness, and whether standard clinical definitions are appropriate for neurofeedback-type interventions. There is, we would argue, a need for standardized assessment of clinical efficacy and effectiveness for feedback-based therapies based on more realistic and appropriate principles.

In this chapter, we ask whether the accepted criteria for appraising evidence about prevention and treatment in general clinical practice can be adequately applied to neurofeedback interventions. While established criteria are useful in evaluating important aspects of the evidence, there are other aspects relevant to neurofeedback that are unique and may not be covered by such criteria. For example, neurofeedback interventions tend to be subtle, complex, long-term, programmatic, and context-dependent. The evidence for their effectiveness must, therefore, be sufficiently sensitive and comprehensive to encompass that complexity. If an intervention is unsuccessful, the evidence should help to determine whether the intervention was ineffective (i.e., a failure of intervention concept or theory), or poorly delivered (i.e., failure of implementation). Clinical interventions are intended to prevent or treat illness in individuals, and while this is still true for neurofeedback interventions, the primary target is typically not the individual or behavior but abnormal physiology in some highly localized area of the brain. Another important difference is the context in which these interventions occur. Neurofeedback interventions are clinic-based (rarely home- or outpatient-based), and contextual characteristics can vary with the type of intervention. Additional difficulties include creating a double-blind condition for an intervention that is grounded on enhancing self-awareness of body and mind. This makes it very difficult to maintain a blind condition when perceptive individuals can quickly recognize that the feedback does not fit with their bodily and mental perceptions. Furthermore, in clinical practice, neurofeedback is often most effective when combined with a wide variety of adjunctive therapies, including relaxation training, visualization, behavior therapies, client education, and other strategies. Finally, and perhaps more relevant to our argument, are the concerns about the relevance of randomized controlled trials (RCT) for evaluating neurofeedback interventions. These concerns include the difficulty of conducting RCTs for complex programmatic interventions, the difficulty in interpreting their results, and the tendency to downgrade the contribution of observational studies [97].

Given these issues, it might be useful to reconsider the criteria for how the effectiveness of feedback training is assessed. We do not want to ignore or deny the need for rigorous examination of the effectiveness and efficacy of these approaches. On the contrary, we propose multi-level criteria based on changes in specific psychophysiological, neuropsychological, behavioral, and community functioning assessments, with proper weight given to these various domains.

## 4. The Need for Objective Outcome Measures

Psychiatrists rely on the classification of symptoms in the Diagnostic and Statistical Manual (DSM), but such classification generally lacks objective measures based on brain function, such as neuroimaging scans, to assist in the diagnosis, treatment selection, and measurement of treatment response in major illnesses. It is now recognized that metrics optimally designed to describe circuit dynamics have the potential to convey important insights for understanding and diagnosing pathophysiology, as well as guiding the development of appropriate treatments.

### 4.1. Psychophysiological Biomarkers

A current assumption in the neurotherapeutic field is that brain changes following neurofeedback (whether functional or structural) underlie clinical improvement. As the view of brain functioning has evolved to one of dynamic and integrative activity across a large number of brain regions, it has been recognized that multiple markers are necessary to give a more complete view of this complex picture, particularly methods that provide an objective analysis of relationships between the different regions involved.

Because frequency-specific brain oscillatory activity can be produced by different neuroanatomical configurations (e.g., 8–12 Hz rhythms in the occipital as well as frontal regions), it is assumed that the same frequency range can reflect different functions. Hence, a biomarker by itself may not be sufficient to differentiate differences in brain processing. Since all brain oscillations are manifested with multiple parameters such as phase-locking enhancement, delay, blocking (desynchronization), and prolongation, it would seem necessary to assess these parameters for a more detailed discrimination of function. A number of researchers have argued that “function is represented in the brain by the superposition of the oscillations in various frequency ranges.” This superposition principle suggests an interdependency and synergy between delta, theta, alpha, beta, and gamma oscillations during the performance of sensory and cognitive tasks [98,99]. Hence, not only are multiple electro-biomarkers and the characterization of their properties necessary, but there needs to be a recognition that these are not totally independent measures. Another biomarker, functional connectivity, has been defined as the temporal correlation of neurophysiological indices measured in different brain areas. As reported by [35], recent studies have increasingly leveraged functional connectivity assays to index the effects of rtfMRI neurofeedback [22,24,35]. However, it appears that changes in functional connectivity may be decoupled from and not necessarily expressed in changes in behavior. Until these relationships are clarified with additional research, we argue that neurofeedback can and should be described as effective at one level (e.g., cellular function) but may or may not translate into effectiveness at another level (e.g., behavior).

### 4.2. Neuropsychological Assessments

Neuropsychological testing provides a level of observation that lies between psychophysiological biomarkers and community functioning. Thus, in evaluating the effectiveness of neurofeedback interventions, in addition to brain-based effects on psychophysiology, we recommend assessment of neuropsychological variables that support cognitive function, which ultimately influences everyday functioning. Cognitive function tests can be organized into broad categories to test intelligence, memory, language, executive function, visuospatial functioning, and disease-specific impairments. Depending on the targets of the feedback, a battery of general tests that assess multiple neuropsychological functions and specific tests can be chosen. For instance, if the feedback is targeted to improve attention, then tests such as the Test of Variables of Attention (TOVA), or Test of Everyday Attention (TEA) would be appropriate. Additionally, we recommend using tests that have shown strong relationships to everyday functioning. The ultimate goal of treatment is to influence and optimize behavior, therefore, behavior and community functioning should also be assessed directly, as discussed in the next section.

### 4.3. Behavioral and Community Functioning

Part of the problem in determining appropriate biomarkers, whether spectral dynamics, functional connectivity, or neuropsychological scores, is that a simplified or reductionist approach, where only a limited aspect of the complexity and dynamic nature of human brain dynamics is characterized, can only give an incomplete view. Importantly, it must be recognized that the brain’s primary role is to optimize the outcome of our behavior while embedded in this ever-changing physical and cognitive context. That means that perhaps the ultimate biomarker of normal functioning is behavioral changes of clinical significance. It is for these reasons that we suggest that a key marker to measure the effectiveness of neurofeedback interventions should be the behavior of the individual in society, i.e., their ability to form relationships and obtain gainful employment or schooling, aspects that lead to successful community functioning. In this regard, studies across multiple disorders have assessed quality of life measures (QoL) directly, or used a proxy in response to NFB training in both adult and pediatric populations. A positive impact on QoL has been noted in children with ADHD [100] and in children with autism spectrum disorders [101]. NFB has shown similar improvements in QoL in methamphetamine-dependent adults compared to a placebo condition [102], in healthy adults undergoing NFB assisted mindfulness-training protocol [103], in multiple sclerosis patients who showed significant reductions in depression and fatigue, both of which are associated with QoL [104], and in traumatic brain injury [105] patients. Beneficial effects on QoL have also been noted in patients with post-cancer cognitive impairment [106], in post-stroke limb rehabilitation [107], and in patients with fibromyalgia [108]. In addition to a continued focus on QoL in response to NFB treatment, we suggest that future studies collect additional measures of community functioning, including the direct impact on the quality of relationships and occupational functioning.

## 5. Structural and Functional Connectivity Studies

We believe that neurofeedback is at a point where the accumulating evidence calls for a re-evaluation of the assessment criteria, including the establishment of standardized and rigorous criteria based on appropriate principles, as outlined above. A brief look at the literature in specific clinical conditions, including autism spectrum disorder, schizophrenia, and psychopathy, provides a compelling reason for this.

### 5.1. Autism Spectrum Disorder (ASD)

In one anatomical MRI study, Hadjikhani and colleagues [109] reported significant cortical thinning in high-functioning adults with ASD compared to matched control participants, particularly in the inferior frontal gyrus (IFG), bilateral inferior parietal lobule (IPL), as well as right superior temporal sulcus (STS). This thinning was correlated with autism symptoms, as diagnosed by the ADI-R [110]. In an fMRI study, Williams and colleagues [111] studied adolescents during finger movement imitation and found reduced BOLD activation for the ASD group in bilateral IPL in comparison with matched typically developing (TD) children. Interestingly, no differences were detected in IFG. On the other hand, Dapretto and colleagues [112] reported reduced BOLD activation in IFG in a study in which they tested boys with ASD during imitation of emotional facial expressions. Although children were able to perform the imitation task, significantly reduced activation in IFG was detected bilaterally in comparison with controls. Villalobos et al. [113] found reduced connectivity between primary visual cortex and bilateral IFG during visuomotor coordination in individuals with ASD, compared to matched TD participants, in a functional connectivity MRI (fcMRI) study. Likewise, studies using resting state-MEG are consistent with the fMRI studies in supporting the notion of dysfunctional connectivity in ASD. Tsiaras et al. [114], for example, showed reduced interdependence strength within bilateral frontal and temporal sensors, as well as between temporal sensors and other recording sites in a group of ASD participants. For all these reasons, it is hypothesized that abnormal functional connections exist that can lead to ineffective neural communication, which in turn impedes early affective, social, and communicative development.

Quantitative EEG (QEEG) findings support an emergent framework similar to the functional connectivity MRI and MEG studies. A number of studies have shown evidence for both global hypoconnectivity and local hyperconnectivity in ASD individuals using phase coherence in multiple frequency bands as a measure of functional connectivity [115,116,117,118]. Several of these studies have noted increased coherence in gamma frequency bands over the parietal [115] and temporal lobes [119], suggesting increased local connectivity. Murias et al. [117] found elevated coherence in the theta (3–6 Hz) frequency range in ASD subjects, primarily over the left frontal and temporal regions, as well as lower coherence in the lower alpha range (8–10 Hz) in the frontal regions. Other studies have shown lower interhemispheric delta and theta coherences across the frontal region, low delta, theta, and beta coherence in posterior regions, and hypocoherence in delta, theta, and alpha frequencies over temporal regions [120]. In contrast, Cornew et al. [121] indicated that children with ASD exhibited regionally specific elevations in delta (1–4 Hz), theta (4–8 Hz), alpha (8–12 Hz), and high-frequency (20–120 Hz) power, supporting an imbalance of neural excitation/inhibition as a neurobiological feature of ASD. We and others have hypothesized that there is a range of overconnectivity as well as underconnectivity in TD children (but more prominent in ASD children) that correlates with varying levels of performance in cognitive, emotional, and behavioral assessments, making them susceptible to improvement via neurofeedback training.

To assess the efficacy of neurofeedback training, Coben and Hudspeth [122] used QEEG-guided NFT to study children with ASD, who showed significantly high levels of mu rhythm activity and a lack of mu suppression during observational activity. Using interhemispheric coherence training designed to increase the connectivity between central and peripheral frontal regions, they showed that groups improved significantly on behavioral and neuropsychological measures. However, only in the coherence training group was mu activity significantly affected, and the increased coherence was associated with improved levels of social functioning. Coben and Padolsky [123] used similar QEEG-guided NFT on ASD patients to reduce hyperconnectivity in posterior-frontal and anterior-temporal regions. Following training, parents reported symptom improvement in 89% of the experimental group, with little change in the control group. Behavioral improvements were reported in attention, visual perceptual functioning, language, and executive functioning, with a 40% reduction in core ASD symptoms as assessed by the Autism Treatment Evaluation Checklist (ATEC) total scores. They also reported decreased hypercoherence in 76% of the experimental group, as measured by a post-training QEEG. The results suggested that decreased hyperconnectivity modulated the positive changes in outcomes.

Altered functional connectivity likely disrupts healthy synchronization and communication among and within neural circuits in the ASD brain, thereby producing changes in processing of sensory inputs necessary for normal social life. Hence, anomalies in functional connectivity could underlie the abnormal social behavior seen in many children with this disorder. Atypical fcMRI and QEEG functional connectivity may be the consequence of early aberrations in white matter development and disturbances in experience-driven network formation through regressive and constructive processes, such as synaptic pruning and stabilization [124]. Although the characterization and specific nature of neural connectivity in the brain is incomplete, the brain’s experience-dependent structural plasticity [125] gives confidence that these abnormal or underfunctioning patterns may be normalized with neurofeedback treatment [126,127]. However, it is reasonable to assume that the efficacy of the training would be assessed differently at different functional levels.

### 5.2. Schizophrenia and Psychopathy

Previous studies suggest that the three most salient structures in the emotional network include the insula, amygdala, and medial prefrontal cortex [128,129,130]. Two studies recently explored the role of the insula in emotional processing in patients with psychopathy and schizophrenia in the context of neurofeedback [85,131]. The studies tested the effects of rtfMRI neurofeedback in promoting healthier function of the insula, a brain structure widely implicated in emotional processing and recognition. In their research, the team found that both left and right insula could be influenced via conscious control using rtfMRI neurofeedback. The results showed altered behavioral tests of facial emotional recognition and simultaneously influenced overall network connectivity, suggesting that feedback influences both neurophysiology- and laboratory-based tests of emotion recognition. The region of interest (ROI) was selected to be the insula, the left side in psychopathy and both sides in schizophrenia, because the insula is associated with cognitive affect regulation.

A group of paranoid subtype schizophrenia patients performed a two-week, four-session rtfMRI BOLD-based neurofeedback training protocol. Each patient had been receiving anti-psychotic medications for at least four weeks prior to the study. As part of their admittance to the study, each subject was evaluated behaviorally using the Positive and Negative Syndrome Scale (PANSS) and the Calgary Depression Scale for Schizophrenia along with informed consent. Changes in connectivity were assessed using a Granger Causal Model (GCM) examining the computed causal density, a metric of temporally directed influences rather than only correlation or coherence. During training, subjects were encouraged—but not instructed—to recall emotionally relevant past experiences as a possible way to modulate the BOLD upregulation on the feedback monitor graphic, a thermometer. On each training day, each subject was re-evaluated based on the Positive and Negative Affect Schedule, and on the final training day all subjects performed a transfer session during which they were asked to self-regulate their insula to observe whether self-regulatory training persisted even without neurofeedback. Results from connectivity analysis in both the schizophrenia and psychopathy studies lend support to the efficaciousness of rtfMRI neurofeedback. Effective connectivity, measured by causal density, was affected by neurofeedback training targeting blood perfusion in the insula, most notably between the insula cortex, amygdala, and medial pre-frontal cortex. It appears that training produced better overall regulatory control (for example “top-down” MidFG to ACC and insula vs. disconnection or “bottom-up” dysregulation) and integrative connectivity between the anterior and posterior regions. The supramarginal gyrus has been widely implicated in empathic perception of others’ emotional state; in the clinical neurofeedback tradition, this area has been known as the tri-state region of the temporal-parietal-occipital (TPO) junction. The area appears to be important for the overall continuity of experience, especially emotional and relational memories [132].

The changes exhibited by schizophrenic patients suggest at least a functional rewiring since Granger causal connections increased overall frontal influence over lower substructures. Further study is warranted to more deeply explore the fundamental mechanisms at play in schizophrenia as well as the possibility of using other modalities like EEG or high-density fNIRS technology to influence insular function. Future work is also warranted regarding a developmental hierarchy of schizophrenia to explore where the dysfunction begins and how it develops into a complex, multi-regional brain disorder.

In the study on emotional processing in psychopathy, patients underwent a similar training regimen, as described previously for schizophrenia patients. However, training targeted BOLD signatures in the left anterior insula rather than both. This ROI was selected based on a localization process in which researchers isolated activity associated with “emotionally relevant personal experiences” by instructing subjects to recall the emotional content of such experiences. Baseline blocks to isolate global background activity were performed before, after, and in between the four emotional imagining blocks, and the background ROI was selected to insulate the left anterior insular ROI from movement and activations from other subcortical emotional networks and regions.

The neurofeedback training included an initial pre-trial, with three days of feedback training four times a day and a final post-trial. For feedback, as in the schizophrenia study, subjects observed an animated thermometer whose level corresponded with the BOLD signature increase over baseline activation at the ROI (BOLD_upreg_—BOLD_baseline_). The protocol design included baseline and upregulation blocks; during the baseline blocks, subjects did not actively attempt to influence the on-screen thermometer and during upregulation blocks the subjects were instructed to increase BOLD response, guided by the thermometer graphic.

Pre- and post-tests were performed as a behavioral measure of change based on volitional regulation of the BOLD signature in the left anterior insula. These used a set point, stimulus, response method in which the subjects alternated between upregulation and baseline neurofeedback, followed by brief presentation of an International Affective Picture System (IAPS) image as stimulus, then response rating block during which participants evaluated their emotional state using the Self-Assessment Manikin test. Of the four subjects trained, one subject learned to increase BOLD activity in the anterior insula with training. Additionally, learning to upregulate the left insula was also associated with increased connection in the emotional brain network.

### 5.3. Depression

A number of recent studies using real-time fMRI and simultaneous EEG and rtfMRI-based neurofeedback have explored if, and how, NFB impacts brain connectivity in patients with depression. To date, previous investigations have suggested that amygdala hemodynamic responses to positive stimuli are attenuated in patients with major depressive disorder (MDD) [133] and that these responses normalize when remission is achieved with antidepressants [134]. Thus, it was hypothesized that NFB-induced increase in amygdala response to recall of happy autobiographical memories would improve symptoms of depression and normalize brain activity. Yuan et al. utilized rtFMRI and NFB to upregulate amygdala activity during recall of happy autobiographical memories (AMs) in a single session in patients with MDD (*n* = 27) and healthy controls (*n* = 27). Subjects received active neurofeedback from the left amygdale (LA) or from the left horizontal segment of the intraparietal sulcus (control region). Pre-/post-resting-state functional connectivity measures showed that abnormal LA hypo-connectivity in MDD was reversed after rtfMRI and NFB training. Additionally, a positive correlation was noted between symptom reduction and connectivity, so that patients with the greatest reduction showed the highest increase in connectivity [135]. In another study, the same group showed that rtfMRI and NFB training to increase amygdala hemodynamic response to positive memories significantly decreased depressive symptoms and increased the percentage of specific positive memories recalled on an autobiographical memory test [136]. An additional study assessed the effects of two sessions of rtfMRI and NFB in patients with MDD who received either amygdala feedback or placebo/control parietal feedback. Here, the investigators assessed pre-/post- effects on emotion processing. Amygdala rtfMRI and NFB training was associated with changes in amygdala responses to happy and sad faces and improved processing of positive stimuli during performance of the Emotional Test Battery [137].

In another study, Hamilton et al. trained depressed patients to downregulate BOLD responses of identified nodes within the salience network (SN) when viewing negative material. Compared to subjects in a sham condition, subjects in the active condition showed greater reduction in SN node response to negative stimuli, indicating changes in brain connectivity. Additionally, greater decreases in responsiveness to negative self-descriptors were also noted in the treated group [86,138] trained eight depressed patients to upregulate BOLD activity in the ventrolateral prefrontal cortex and insula during four rtfMRI and NFB sessions. Subjects showed reduced symptom burden and evidence of increased bilateral activations in the areas trained (anterior insula, VLPFC), but also in other areas including parts of the hippocampus, right ventral striatum, and left cuneus, as well as deactivation at the temporo-parietal junction, posterior insula, and the right dorsolateral prefrontal cortex (DLPFC), indicating network-level changes.

In another study, Zotev et al. trained MDD patients (*n* = 13) to upregulate BOLD activity of the left amygdala using an rtfMRI-NFB during a happy emotion induction task, while MDD patients in the control group (*n* = 11) were trained to sham rtfMRI and NFB. Simultaneous scalp EEG recordings were collected during training to assess the relationship between NFB, frontal asymmetry in the upper alpha band (FA-alpha), which has been associated with depression, and BOLD activity in regions implicated in emotion regulation. The results showed that FA-alpha was reduced, while BOLD activity in the amygdala was enhanced during rtfMRI and NFB, thus demonstrating NFBs; effects on both neural change and electrical readouts from the scalp. Conversely, EEG and NFB-induced reduction of frontal alpha asymmetry is postulated to improve connectivity in neural networks that support emotion regulation [139].

In summary, there is growing evidence that supports the positive effects of NFB on depression. These positive effects are accompanied by both behavioral changes (patient self-reported and clinician-administered tasks), as well as neural changes, as measured through fMRI BOLD activity and scalp EEG recordings, and are consistent with changes observed in patients treated with other modalities such as antidepressants.

## 6. Conclusions

There is increasing optimism and interest in the general population about neurotherapeutic interventions, such as neurofeedback and biofeedback, which give individuals a more active role in their own health care, utilize a holistic approach to body, mind, and spirit, are non-invasive, and elicit the body’s own healing response [140,141]. This renewed interest is coupled with an array of methodologies that allow for neurofeedback of brain electromagnetic activity as well as blood flow.

In contrast to this growing enthusiasm, there is ample skepticism about the effectiveness of these methodologies. Many agree that for neurofeedback to earn acceptance as a valid intervention, the clinical significance of its purported therapeutic effects must be clearly established [35]. First and foremost, proper control conditions are necessary, including sham conditions. Additionally, better ways to separate non-specific factors from the feedback itself, including control strategies, expectations, attentional factors, etc. is also important. In addition to conducting more rigorous studies, we are also suggesting an open discussion of optimal methods to measure the effects of neurofeedback and biofeedback approaches. Until more placebo-controlled, randomized trials are available, it seems reasonable to assess NFBs’ effects at various levels (biological, neuropsychological, and behavioral) and assess how well the interventions work within these acknowledged semi-independent levels of processing. Neuroplasticity, as the medium of the brain that is affected by neurotherapeutic interventions, immediately suggests that changes will occur at the level of gene expression and behavior, as well as everything in between. We suggest that brain changes occasioned by specific neurofeedback protocols will be reflected at one level by some biomarkers and not others. Because of the semi-independent relationship between levels, and differential time courses, changes in physiology may become apparent early in treatment, whereas behavioral changes may take longer to manifest. Additionally, as is true of most treatments of neuropsychiatric disorders, studies that focus heavily on group differences may not be sufficient to determine whether treatment is effective in some patients but not others. Given these limitations, we suggest a strategy that assesses efficacy at multiple levels and examines data for each subject, as previously suggested by Samuel et al. using an N-of-1 study approach, in order to usher in the era of personalized medicine [142]. We hope that taking such an approach will shed more light on neurofeedback’s utility as a viable treatment for neuropsychiatric disorders.

Returning to the possibility of using NFT for the purposes and goals of social neuroscience, it seems that experimental neurofeedback designs with specific behavioral outcomes would inform both the social neuroscience and clinical communities. Some markers of neural activation have been relatively well studied and may serve to pioneer such methodology in social neuroscience. For instance, markers of attention, focus, memory, and emotion regulation may be considered manipulated interventions in which social outcomes might be measured. To provide a concrete example, social neuroscience networks associated with mirroring others’ behaviors are thought to provide understanding of others’ movements, and this information is thought to inform mentalizing systems in which intention understanding takes place [143]. Such networks have been manipulated in NFT treatment of individuals with autism in the hopes of engaging such networks for better mirroring and subsequent mentalizing function [2]. Pineda and colleagues found greater activation of such mirroring systems when participants viewed social interactions for participants in the experimental but not the sham condition. To bridge these two fields, experimental work in social neuroscience might investigate participants’ tendencies or abilities in mentalizing and mirroring following NFT training thought to engage such systems. This is only one of many possible applications of NFT that has the potential to cross disciplines. Here we have provided an overview and framework through which to consider the complexities and application of such methodology. Neurofeedback may be a useful tool in our understanding of neural processes, be that for the goal of treatment or understanding.
